# Nursing Care Plan for Patients with Hajdu–Cheney Syndrome

**DOI:** 10.3390/ijerph19127489

**Published:** 2022-06-18

**Authors:** Jonathan Cortés-Martín, Lourdes Díaz-Rodríguez, Beatriz Piqueras-Sola, Juan Carlos Sánchez-García, María José Menor-Rodríguez, Raquel Rodríguez-Blanque

**Affiliations:** 1 Research Group CTS1068, Andalusia Research Plan, Junta de Andalucía, 18014 Granada, Spain; jcortesmartin@ugr.es (J.C.-M.); cldiaz@ugr.es (L.D.-R.); bpiquerassola@gmail.com (B.P.-S.); rarobladoc@ugr.es (R.R.-B.); 2 Faculty of Health Sciences, School of Nursing, University of Granada, 18071 Granada, Spain; 3 Hospital University Virgen de las Nieves, 18014 Granada, Spain; 4 Subdirectora de Humanización y Atención al Ciudadano, Área Sanitaria Santiago-Barbanza, 15706 Santiago de Compostela, Spain; mariajosemenor@hotmail.com

**Keywords:** rare disease, Hajdu–Cheney syndrome, nursing care plan, acroosteolysis, NOTCH2, clinical practice, healthcare

## Abstract

Hajdu–Cheney syndrome is a rare genetic disease. Its main features include phenotypic variability, age-dependent progression and the presence of acroosteolysis of the distal phalanges and generalized osteoporosis, which have significant disabling potential. Currently, there is no effective curative treatment, so nursing care is essential to ensure the maintenance of the quality of life of these patients. The main objective of this study was to establish a specific standardized nursing care plan using the NANDA–NIC–NOC taxonomy. The application of a care plan as such would improve the quality of life of patients affected by this rare disease, will contribute to increasing healthcare professionals’ knowledge on this matter and will support future studies on this disease.

## 1. Introduction

To date, more than 7000 rare diseases have been described around the world, yet very little scientific knowledge has been generated for only about 800 of them. Eighty percent of rare diseases have a genetic origin and have high mortality rates [1]. By definition, rare diseases have a low prevalence in the general population, and Hajdu–Cheney syndrome (HCS) specifically has very few reported cases [2]. These cases are presented in the context of limited and dispersed study samples, with variable phenotypes and clinical manifestations that have not been clearly documented and show different clinical courses [3].

Considering the clinical and scientific panorama where this disease makes its appearance, it is important to note that standardization and universalization of specific practices and diagnostic tests would simplify the workflow and significantly contribute to the advancement of research. A detailed description of cases would contribute to reducing the time to diagnosis, improve the quality of treatments and offer better overall assistance to each patient. An update of the cases already reported would be of great help to carry out a cross-sectional study from a new standpoint, offering a better, more global perspective of this disease. For this reason, a closer consideration of the phenotypic differences, the clinical presentation and courses the disease takes in each patient and the development of a specific intervention plan for HCS patients would make the management of this disease more effective and straightforward [4].

Currently, there is no effective curative treatment for this disease, so nursing care is ever more important to ensure the maintenance of the quality of life and well-being of these patients, their families, caregivers and friends.

There are no scientific publications that focus solely on nursing care and the role of nurses in the care of patients diagnosed with Hajdu–Cheney syndrome. A specific standardized nursing care plan for this disorder will update knowledge in this field and will contribute to an improvement in quality of care by enabling better management of the disease, leading to an improvement in the quality of life of these patients and their families.

### Background

Hajdu–Cheney syndrome was first described by N. Hajdu in 1948 [5], and the description was completed at a later date by D. Cheney in 1965 [6]. Since then, approximately 100 cases have been reported worldwide, which has led to the identification of the three main features that are shared by all patients: phenotypic variability [7], age-dependent progression [3] and the presence of generalized osteoporosis and acroosteolysis of the distal phalanges as well as other clinical manifestations [8].

This syndrome is classified as a rare genetic disease, with classification references ORPHA955 in the ORPHANET database [2] and #102500 in the OMIM database [9]. It mainly affects the connective tissue and belongs to the osteolysis syndromes group [10].

It is caused by a heterozygotic mutation of the gene NOTCH2 [11] located on chromosome 1p13-p11. This gene is closely linked to skeletal development [12], so alterations at this level will lead to skeletal disorders. This disease follows an autosomal-dominant inheritance pattern [13], although descriptions of cases with sporadic mutations can be found [14]. As in many other rare diseases, the prevalence of HCS is less than one in one million live births (<1/1,000,000) [2].

A definitive diagnosis is reached by genetic sequencing, although the initial diagnosis is suspected based on the observation of phenotype and radiological findings [15]. Due to the phenotypic variability, on occasion, other syndromes may have to be included in the differential diagnosis as there are certain overlapping features with diseases such as Alagille syndrome or lateral meningocele [16,17].

Considering the degenerative nature and the phenotypic variability of this disease, it is practically impossible to observe the complete and definitive phenotype of the disease in a single person. The clinical signs and symptoms appear in different bodily systems. Some of the frequently found clinical manifestations include cranial and facial alterations [5], premature denture loss [18], short stature [19], joint laxity, cervical instability [20], osteolysis of the distal phalanges and generalized osteoporosis [21]. Other clinical manifestations may include cardiovascular alterations [22], renal impairment [23], delayed motor development and hearing and sight loss [24], among others.

Some of the clinical complications that have been found in this syndrome include basilar invagination [5], hydrocephalus [25], vertebral collapse due to compression [26] and ventilatory restriction due to thoracic deformities [27].

Hajdu–Cheney syndrome is classified as a severe rare disease; however, no studies offer a global perspective of the disease regarding prognosis and quality of life of the affected patients. The severity of the disease depends on the organs and systems that are affected, the clinical complications and the degenerative progression of the disease in each patient. The generalized osteoporosis and the development of acroosteolysis are responsible for fractures, difficulties in walking and dependency for activities of daily living.

Prognosis worsens when complications such as basilar invagination exist, causing neurologic alterations or thoracic deformities that cause ventilatory restriction. Due to the low prevalence of HCS and the scarcity of qualitative information on this syndrome, it is difficult to determine the burden of disease and years of healthy life lost.

Currently, there is no definitive or effective treatment for HCS. Studies on pharmacological treatments for this disease show no clear evidence on their efficacy. Bisphosphates are the drug group of choice in certain cases [28,29], whereas surgical treatment generally aims to alleviate certain clinical findings and complications [30].

As there is no definitive treatment for this disease, management of this syndrome focuses on offering the best care available to improve the quality of life and life expectancy of these patients.

Nursing professionals are responsible for offering evidence-based quality care in order to guarantee the well-being of these patients and their families. This fact relates to the objective of this study, considering that no nursing care plans that standardize care for these patients have been developed to date. Having a plan of such characteristics would be of great help for nursing practice, for health education and for better management of this disease.

The main objective of this article is to present a standardized care plan specifically for patients diagnosed with Hajdu–Cheney syndrome that will impact the approach in management and care in this disease and, therefore, the quality of life of these patients and their families.

## 2. Materials and Methods

Throughout the development of this report, nursing methodology was consistently applied—in other words, a direct application of the scientific method of nursing care [31]. It is a systematic process known as the “Nursing Care Process” [32], which establishes a framework for offering rational, logical and efficient care by focusing on achieving expected outcomes by means of a series of interventions. It is an organized method that is classified as a deductive theory as of itself and is considered a quality standard in professional practice. Therefore, the nursing profession is a legitimate scientific discipline [33].

This standardized nursing care plan for Hajdu–Cheney syndrome has been developed following the guideline of the “Nursing Care Process”. It aims to ensure the quality of the care and provides the basis for operational control and the means for systemizing and performing research in this field. The process outlines five phases: evaluation, diagnosis, planification, execution and assessment [31]. By applying this method, the needs of each patient will be detected, problems will be identified and solutions will be proposed by offering evidence-based nursing care. Once problems have been identified, concrete goals and outcomes should be set, alongside the proposal and planification of specific interventions to aid in reaching said goals.

It is necessary to emphasize that this report presents a standard nursing care plan for patients diagnosed with Hajdu–Cheney syndrome. Therefore, before its implementation, it is essential to adapt and personalize each of the five phases that make up the care plan for the specific patient. The development of the last two phases, execution and assessment, will depend directly on the implementation of the plan, being different for each specific patient.

### 2.1. Evaluation

Evaluation is the first phase of the Nursing Care Process. In this phase, the basic needs of the patient, their family and their environment are determined, information which is essential in establishing a problems diagnosis [32].

The evaluation established in this nursing care plan was developed following the system designed by Marjory Gordon [34]. It is a system the fulfills all the criteria needed to carry out an effective nursing evaluation, so it constitutes a useful evaluation tool in any nursing discipline module. It defines eleven functional patterns as established behaviors that are shared by all human beings and which contribute to their health and quality of life over time and independent of age and disorders.

In order to detect needs in this phase, a review of the literature [4] was carried out with the aim of obtaining a complete knowledge of the syndrome. In total, 115 described cases published from 1948 to 2022 were analyzed, and 12 patients with a confirmed diagnosis were contacted directly [35].

### 2.2. Diagnosis

During the diagnosis phase, the problems that arise from the specific needs are identified. Considering how the different functional patterns are affected and using the NANDA [36] taxonomy, a classification into real or potential problems is made, as well as differentiating problems relating to autonomy, nursing diagnoses and collaboration issues [37].

### 2.3. Planification

Once the problems have been identified, a care plan is developed with the aim of providing solutions to the problems. Standard outcomes are established which will be reached by means of a series of interventions. In this case, the guidelines followed for the writing of the objectives and interventions are the NOC [38] and NIC [39] taxonomies.

In addition to the previously mentioned tools used in the development of this report, we used the NNNConsult platform [40].

The organization and sequencing of the Nursing Care Process are essential for the correct development of a nursing care plan. As we pointed out earlier, this report presents a series of indications for a standardized care plan that must be adapted and tailored to each specific case before it can be implemented.

This report was presented to and gained approval from the Research Ethics Committee of the Province of Granada (Spain) in 2 March 2021.

### 2.4. Execution and Assessment

Both phases of the care plan must be analyzed once it has been implemented with a specific patient.

## 3. Results

### 3.1. Evaluation

The evaluation is shown in the following table (Table 1) and was designed according to the functional patterns described by Marjory Gordon [34]. In this evaluation, a series of different patterns are presented alongside specific observations regarding the disorder in question and a list of useful nursing scales for quantifying the evaluations.

### 3.2. Nursing Care Plan

We now present the standardized specific nursing care plan for Hajdu–Cheney syndrome. Three types of problems are distinguished: autonomy problems, collaboration problems and nursing problems, reported according to the NANDA taxonomy, also known as a nursing diagnosis. Additionally, we refer to real or potential issues in each segment [37].

#### 3.2.1. Nursing Diagnosis

The problems that are detected which are a concern for nurses are commonly referred to as a nursing diagnosis [31]. In Appendix A, we present the nursing diagnosis along with the expected outcomes (NOC) [38] after the implementation of specific interventions (NIC) [39] by means of different activities. Additionally, the diagnoses are organized according to the functional patterns used for the nursing evaluation. The distinction between real problems and potential problems is implicit in the table in the appendix.

As can be seen in Appendix A, most of the problems detected in this section are related to impaired mobility and the high disabling potential of the syndrome, which has a negative impact on other aspects. Pain is another important factor to highlight as it directly influences the development of daily routine. Furthermore, one of the main complications of this syndrome is problems related to breathing, caused by chest deformity. It is also worth mentioning the fear and anxiety in a situation of uncertainty about the health situation in the future.

#### 3.2.2. Autonomy Problems

Autonomy problems are problems in which the independence of the patient is compromised [33]. These issues reveal a total or partial deficit in the physical or psychological ability of the patient to carry out the required actions to satisfy their needs. These problems may be temporary or permanent, but they are always classified as real. In Appendix B, we present the autonomy problems related to the interventions and nursing-specific activities following the NIC taxonomy [39].

As can be seen in Appendix B, the high disabling potential and degenerative nature of this pathology have a negative impact on the autonomy of these patients. Autonomy can compromise aspects such as feeding, elimination, mobilization and personal hygiene, among others.

#### 3.2.3. Collaboration Problems

Collaboration problems are health problems in which the patient needs nurses to carry out specific control- or treatment-related activities that were prescribed by another healthcare provider [31]. This type of issue can be classified as real or potential. In Appendix C, we present specific collaboration issues that arise in this syndrome related to the interventions and nursing-specific activities following the NOC taxonomy [39].

Appendix C confirms that a multidisciplinary perspective is necessary to care for this type of patient. The relationships between nursing and other health fields such as medicine, psychology, physiotherapy and podiatry are evident in this section. All of them are essential for the care of patients diagnosed with this syndrome.

## 4. Discussion

It is of utmost importance to understand the scenario in which this disease develops so as to fully comprehend the analysis of this report from the correct perspective.

Although advances in the search for treatments, both pharmacological [28,29,60] and surgical [20,30], have been remarkable in recent years, there is still no effective curative treatment for this syndrome to date, thus highlighting the need for this report.

It is worth noting that what we present is a standardized nursing care plan, so before implementation, it must be personalized to adapt it to the specific needs of each patient and altered, if necessary, after each intervention.

The main features of this syndrome stand out after the initial evaluation. The degenerative and invalidating nature of the disease, as described by Jirecjová et al. [61] and Brennan et al. [3] in their publications, highly impacts patients’ autonomy and increases their levels of dependence. The generalized osteoporosis and osteolysis of the distal phalanges, reported by numerous experts on the matter such as Brown et al. [8] and Rosenmann et al. [21], noticeably limit mobility and reduce the possibilities of walking without assistance. The general uncertainty and scarcity of knowledge that come with any rare disease generate a state of anxiety and stress that affects mental health [62].

The perception and management of health in these patients is closely related to their level of autonomy. The invalidating nature of the disorder, previously described by Descartes et al. [14] in their studies, is the reason behind the fact that most patients require assistance for everyday life activities. It is of utmost importance to highlight the role of carers and their relationship with the patient and their families and note that it is a responsibility of nursing to also care for carers. Respiratory infections, such as those presented in the cases reported by Williams et al. [63] and Sasaki et al. [64], as well as urinary tract infections, such as those described in the case report by Currarino et al. [23], are common complications, so patients and their families must be instructed on how to prevent and manage them. Mobility issues are closely linked to risk of falls, so it is important to implement exercises that work on balance, gait control and the use of walking assistance devices or mobility aids. These issues have been broadly discussed in several articles, including one by Colmenares et al. [65]. In patients who have very limited mobility and who spend long hours in bed, there is a high probability that pressure ulcers will develop if no measures are taken to prevent them. Education on pressure ulcer prevention is key, including frequent control of high-pressure areas, postural changes and the use of anti-bedsore air mattresses.

The premature loss of teeth, described in publications by Lee et al. [66] and Shaw et al. [18], as well as possible intestinal malrotation, as Hajdu [5] reported in his first description of the syndrome, are problems that generate nutritional issues and complicate management. In these cases, malnutrition must be sought and ruled out. Nursing care practices must include detection of the risk of malnutrition and education regarding healthy eating habits and oral care and hygiene. These points are discussed in depth by Vigerhoedt et al. in their study [67].

With regards to elimination, constipation is one of the most frequent symptoms in patients with HCS. Encouraging physical activity and implementing a healthy balanced diet are key aspects in constipation prevention. In more severe cases, the use of laxatives or other drugs that increase intestinal motility may be required, as is reported in some of the cases described in the literature [35].

There is a group of patients who present with a clinical combination that is characteristic of this syndrome: polycystic kidneys and serpentine fibula. In these patients, due to the multiple cysts in their kidneys, alterations in urinary elimination may appear. Fryns et al. [68] and Ramos et al. [69] made interesting contributions on this matter.

Some of the main problems of this disease are the generalized osteoporosis and the osteolysis of the distal phalanges that develop and worsen over time [8]. Alterations in bone structures have an effect on mobility at all levels, contributing to the loss of autonomy and increasing the risk of injuries and falls, as reported by Greenberg et al. [70]. Thoracic bone deformities may limit the ventilation of basal sectors and increase the risk of infections. Extreme fatigue with minimum efforts, tiredness and strength loss also translate into limited periods of time dedicated to physical exercise.

The sleep pattern in these patients is also often affected, and many require the administration of medication to aid in falling asleep [35].

Pain management is another main issue that must be tackled by nurses treating this disorder. This issue is highlighted by Brown et al. [8] and Harnasch [71]. The identification and measurement of pain are essential for adequate pain management. Postural changes and the use of painkillers are effective tools in these situations.

The emotional state of a patient diagnosed with HCS is often related to their disease process. Anxiety and stress arise from uncertainty and fear of the future [62]. Creating safe spaces where the patients and their families can talk and share their thoughts and feelings is useful in disorders such as this. Emotional support is very important for these patients. The stress caused by the disease burden must be treated, including specific care regimes that favor family communication and facing of reality.

Social relationships often suffer due to communication issues. Improving speech clarity and hearing loss are nursing care issues that must be taken into account. Herrman et al. [24] described a case with hearing difficulties that highlights the importance of this kind of care.

Sexuality need not be affected in these cases; however, it is important to identify possible risks in the reproductive function of women affected by HCS, especially if complications have already been diagnosed, as in the case of the patient reported by Nozaki et al. [72].

Aspects relating to values and beliefs are very personal matters and difficult to consider in a standardized way.

It is worth noting once again that the nursing care plan we have proposed constitutes a standardized nursing care plan for this syndrome which must be personalized and adapted to each patient, their family and their environment before implementing it in clinical practice.

The scarcity of scientific publications and knowledge on this syndrome as well as its low prevalence are the main limitations found during the development of this study.

## 5. Conclusions

Prior to this study, the nursing care and management of this syndrome was lacking specific indications for the care of HCS patients, their families and their environment. The implementation of this nursing care plan would imply an advancement in the knowledge of this syndrome and an improvement in the well-being of patients. The universalization and standardization of nursing care for Hajdu–Cheney syndrome will serve as a firm basis of knowledge on which to build future lines of research on this matter.

## 6. Limitations

The limitations of this study are related to the low prevalence of the disease and, consequently, to the difficulty in finding a large population sample.

Occasionally, due to the specificity of the syndrome, complications arose regarding the use of the NANDA–NOC–NIC taxonomy. Another limitation encountered in conducting this study is the scarcity of scientific publications on the subject.

## Figures and Tables

**Table 1:** Evaluation according to Marjory Gordon functional patterns.

Functional Patterns	Observations	Proposed Scores and Scales
Pattern 1: Health perception—health management	The context of a patient diagnosed with HCS with regards to perception and health management is abnormal. Due to the high potential of disability that accompanies this syndrome, there is a deficit in autonomy in the maintenance of healthy habits involving personal hygiene and cleaning of the home. HCS patients require the help of third parties. The scarcity of knowledge surrounding the disease, delays in diagnosis and the absence of an effective treatment negatively impact the individual’s perception of health. The risk of accidents, either work- or traffic-related or at home, is patent due to difficulties in walking without assistance. Numerous hospital admissions impact the alteration of this functional pattern.	-Barber [41] (risk of dependence) -Fall risk index [42] (risk of falls) -Tinetti [43] (static balance and gait balance) -Goldberg Ghq28 [44] (general health)
Pattern 2: Nutritional—metabolic	There are problems with eating due to the premature loss of dental pieces and the presence of cavities. Another factor that complicates feeding is intestinal malrotation that may be present in some patients. In certain cases, different food allergies may appear. Short stature is another clinical manifestation of this syndrome. Evaluation of skin may be abnormal as certain patients may have plantar ulcers, and HCS patients’ nails are characteristically short and bulky. A generalized hirsutism may be present.	-MUST [45] (risk of malnutrition) -Norton [46] (risk of pressure ulcers) -Braden [46] (risk of pressure ulcers)
Pattern 3: Elimination	The prevalence of constipation is high in HCS patients, often requiring the use of laxatives. The presence of small polycystic kidneys limits urinary clearance. Urinary tract infections are frequent. The use of absorbent pads or diapers is common considering limited mobility issues.	-Bristol scale [47] (consistency of stools) -Bonney test [48] (urinary incontinence)
Pattern 4: Activity—exercise	Generalized osteoporosis and skeletal malformations limit mobility. Thoracic deformities impede normal ventilation. Excessive weakness. Fatigue with minimal efforts. Dependency for activities of daily living. In some cases, there are congenital heart defects and septal defects. Recurrent respiratory infections. High risk of falls due to instability when standing.	-Barthel [49] (functional assessment) -Katz [50] (autonomy for activities of daily living) -Karnofski [51] (quality of life) -Pain Visual Analog Scale (VAS) [52] (pain intensity)
Pattern 5: Sleep—rest	Chronic pain is present in all patients diagnosed with HCS, which affects falling asleep if uncontrolled. Anxiety and depression are common psychological disorders in HCS patients. The use of sleeping pills is frequent to aid falling asleep and sleep maintenance.	-Oviedo [53] (level of sleep satisfaction)
Pattern 6: Cognitive—perceptual	Delay in speech and language acquisition. Perceptive alterations such as hypoacusis and progressive vision loss. Acute pain and chronic invalidating pain. Depression.	-Pfeiffer [54] (cognitive decline) -Glasgow [55] (level of consciousness)
Pattern 7: Self-perception—self-concept	Deep voice. Limited physical abilities. Altered postural and mobility patterns.	-Gardner [56] (body image)
Pattern 8: Role—relationships	Family relationships are affected by dependency. The adaption to different scenarios may cause social rejection.	-Duke-Unc [57] (perceived social support) -Zarit [58] (carer burnout)
Pattern 9: Sexuality and reproductive	In certain cases, issues may arise during women’s reproductive stage.	-
Pattern 10: Coping—stress tolerance	Stress is present in the majority of these patients due to uncertainties about the future and the numerous hospital admissions.	-Perceived stress scale [59] (stress levels)
Pattern 11: Values—beliefs	There are concerns regarding the meaning of life, death, pain and illness.	-

## Data Availability

Data are available upon request from the corresponding author.

## Appendix A. Nursing Diagnosis

**Pattern 1: Perception—Health Management**			
**NANDA**	**NOC**	**NIC**	**ACTIVITIES**
[00004] Risk of infection	[0703] Severity of infection [1902] Risk Control	[6540] Infection Control	-Teach caregivers proper handwashing -Instruct the patient on the correct hand washing techniques. -Administer antibiotic treatment when appropriate. -Teach the patient and family to avoid infections.
[00035] Risk of injury	[1912] Falls [1910] Safe Home Environment [0200] Ambulate [1828] Knowledge: Fall Prevention [1926] Safe Wandering [0202] Balance [0208] Mobility [0212] Coordinated Movement	[6490] Fall Prevention [3520] Care of pressure ulcers	-Identify cognitive or physical deficits of the patient that may increase the possibility of falls in a given environment. -Control gait, balance and fatigue when walking. -Teach the patient how to fall to minimize the risk of injury -Use an established risk assessment tool to assess the individual’s risk factors (Braden scale). -Closely monitor any reddened area. -Apply protective barriers, such as absorbent creams or compresses, to remove excess moisture, as appropriate. -Inspect the skin of bony prominences and other pressure points when changing position at least once a day. -Apply protectors for the elbows and heels, as appropriate. -Teach family members/caregiver to watch for signs of skin breaks, as appropriate.
[00036] Choking Hazard	[0403] Respiratory Status: Ventilation	[3140] Airway management [3350] Respiratory Monitoring	-Position the patient to maximize ventilation potential. -Perform chest physiotherapy, if indicated. -Remove secretions by encouraging coughing or by suction. -Teach the patient to use the prescribed inhalers, if applicable. Use fun techniques to stimulate deep breathing in children (make soap bubbles; blow a whistle, harmonica, balloons; have a contest blowing ping-pong balls, feathers, etc.). -Monitor respiratory status and oxygenation, as appropriate. -Monitor the frequency, rhythm, depth and effort of the breaths. -Evaluate chest movement, observing symmetry, use of accessory muscles and intercostal and supraclavicular muscle retractions. -Watch for noisy breathing, such as stridor or snoring
**Pattern 2: Nutritional—Metabolic**			
**NANDA**	**NOC**	**NIC**	**ACTIVITIES**
[00002] Nutritional imbalance: lower than body needs	[1100] Oral Health [0303] Self-care: eating	[1100] Nutrition Management	-Determine the nutritional status of the patient and their ability to meet nutritional needs. -Identify the patient’s food allergies or intolerances. -Instruct the patient on nutritional needs (i.e., discuss dietary guidelines and food pyramids).
[00046] Impaired skin integrity	[1101] Tissue Integrity: Skin and Mucous Membranes	[3520] Care of pressure ulcers [3590] Skin Watch [840] Position Change [1660] Foot care [940] Traction/Immobilization Care	-Use an established risk assessment tool to assess the individual’s risk factors (Braden scale). -Closely monitor any reddened area. -Apply protective barriers, such as absorbent creams or compresses, to remove excess moisture, as appropriate. -Place on a suitable therapeutic mattress/bed. -Place in the specified therapeutic position. -Instruct the patient/family on the importance of foot care. -Cut toenails of normal thickness when they are soft, with a nail clipper and using the curve of the finger as a guide. -Refer to the podiatrist to cut thick nails, as appropriate.
[00047] Risk of deterioration of skin integrity	[1902] Risk Control	[3540] Prevention of pressure ulcers [3590] Skin Watch	-Use an established risk assessment tool to assess the individual’s risk factors (Braden scale). -Closely monitor any reddened areas.
[00048] Deterioration of the dentition	[1100] Oral Health [0308] Self-care: oral hygiene	[1710] Maintenance of oral health [1730] Restoration of oral health [5510] Health education	-Establish an oral care routine. -Identify the risk of developing stomatitis secondary to drug therapy. -Teach the person to brush their teeth, gums and tongue.
[00197] Risk of dysfunctional gastrointestinal motility	[0501] Intestinal elimination [1902] Risk Control	[200] Promotion of exercise [6650] Surveillance	-Determine the individual’s motivation to start/continue with the exercise program. -Explore obstacles to exercise. -Help the individual to establish the short and long term goals of the exercise program. -Monitor the individual’s response to the exercise program.
[00315] Delayed infant motor development	[0208] Mobility [1308] Adaptation to physical disability	[6490] Fall Prevention [6650] Surveillance [200] Promotion of exercise	-Identify cognitive or physical deficits of the patient that may increase the possibility of falls in a given environment. -Control gait, balance and fatigue when walking. -Monitor the individual’s response to the exercise program. -Determine the individual’s motivation to start/continue with the exercise program.
**Pattern 3: Elimination**			
**NANDA**	**NOC**	**NIC**	**ACTIVITIES**
[00011] Constipation	[0501] Intestinal elimination [2102] Pain Level [1621] Adherence behavior: healthy diet [0208] Mobility	[450] Management of constipation/faecal impaction [466] Enema Administration [200] Promotion of exercise	-Monitor the appearance of signs and symptoms of constipation. -Identify the factors (medications, bed rest and diet) that can cause or contribute to constipation. Administer enema or irrigation, when appropriate. -Determine the individual’s motivation to start/continue with the exercise program.
[00016] Impaired urinary elimination	[0503] Urinary elimination [1608] Symptom Control	[590] Management of urinary elimination [6540] Infection Control	-Observe for signs and symptoms of urinary retention. -Identify the factors that contribute to episodes of incontinence. -Explain to the patient the signs and symptoms of urinary tract infection. -Teach caregivers proper handwashing -Instruct the patient on the correct hand washing techniques. -Administer antibiotic treatment when appropriate. -Teach the patient and family to avoid infections.
**Pattern 4: Activity—Exercise**			
**NANDA**	**NOC**	**NIC**	**ACTIVITIES**
[00032] Ineffective breathing pattern	[0403] Respiratory Status: Ventilation	[3140] Airway management [3350] Respiratory Monitoring [3320] Oxygen therapy	-Position the patient to maximize ventilation potential. -Perform chest physiotherapy, if indicated. -Remove secretions by encouraging coughing or by suction. -Teach the patient to use the prescribed inhalers, if applicable. Use fun techniques to stimulate deep breathing in children (make soap bubbles; blow a whistle, harmonica, balloons; have a contest blowing ping-pong balls, feathers, etc.). -Monitor respiratory status and oxygenation, as appropriate. -Monitor the frequency, rhythm, depth and effort of the breaths. -Evaluate chest movement, observing symmetry, use of accessory muscles and intercostal and supraclavicular muscle retractions. -Observe if noisy breathing occurs, such as stridor or snoring. -Administer supplemental oxygen as ordered. -Monitor the flow of liters of oxygen.
[00033] Impaired spontaneous ventilation	[0403] Respiratory Status: Ventilation	[3390] Ventilation Aid [3350] Respiratory Monitoring [6650] Surveillance	-Monitor respiratory status and oxygenation, as appropriate. -Administer supplemental oxygen as ordered.
[00085] Impairment of physical mobility	[0200] Ambulate [0201] Ambular: wheelchair [0208] Mobility [1308] Adaptation to physical disability [0202] Balance [0206] Joint movement [0210] Perform Transfer [3110] Self-monitoring: osteoporosis [2102] Pain Level	[221] Exercise therapy: ambulation [1805] Help with self-care: aivd [1806] Help with self-care: transfer [200] Promotion of exercise [222] Exercise Therapy: Balance [6490] Fall Prevention	-Teach the patient to get into the correct position during the transfer process. -Assist the patient with the initial ambulation, if necessary. -Instruct the patient/caregiver about safe transfer and ambulation techniques. -Observe the patient’s need for adapted devices for personal hygiene, dressing, personal grooming, grooming and eating. -Help the patient to accept dependency needs. -Control gait, balance and fatigue when walking.
[00093] Fatigue	[0003] Rest [1209] Motivation [0005] Activity Tolerance [2004] Physical Form	[200] Promotion of exercise [221] Exercise therapy: ambulation [226] Exercise Therapy: Muscle Control [222] Exercise Therapy: Balance [224] Exercise Therapy: Joint Mobility [6040] Relaxation therapy	-Assist the patient with the initial ambulation, if necessary. -Instruct the patient/caregiver about safe transfer and ambulation techniques. -Control gait, balance and fatigue when walking. -Determine the limitations of joint movement and its effect on function. -Protect the patient from trauma during exercise. -Create a quiet environment, without interruptions, with soft lights and a comfortable temperature, when possible.
[00102] Food self-care deficit	[0303] Self-care: eating [1308] Adaptation to physical disability	[1803] Help with self-care: feeding [1100] Nutrition Management	-Provide social interaction, as appropriate. -Provide devices adapted to facilitate self-feeding (long handles, handles with a large circumference, or small straps on utensils), if necessary. -Place the patient in a comfortable position. -Instruct the patient on nutritional needs (i.e., discuss dietary guidelines and food pyramids).
[00108] Self-care deficit in the bathroom	[0301] Self-care: bath [0305] Self-care: hygiene [0208] Mobility [1308] Adaptation to physical disability	[1801] Help with self-care: bathing/hygiene	-Provide a therapeutic environment that guarantees a warm, relaxing, private and personalized experience. -Facilitate the maintenance of the patient’s routines at bedtime, signs of sleep onset and familiar objects (for children their favorite blanket or toy, rocking, pacifier or story; for adults read a book or have a pillow from home), as appropriate.
[00109] Self-care deficit in clothing	[0302] Self-care: dressing	[1630] Dress [1802] Help with self-care: dressing/grooming	-Be available to help with dressing, if needed. -Make it easier for the patient to comb their hair, if that is the case. -Encourage the patient to shave himself, as appropriate. -Maintain privacy when the patient is dressed.
[00110] Self-care deficit in the use of the toilet	[0310] Self-care: toilet use [0202] Balance [0208] Mobility	[1804] Help with self-care: urination/defecation [5606] Teaching: individual [1800] Help with self-care	-Provide privacy during elimination. -Facilitate hygiene after urinating / defecating after finishing elimination. -Provide assistive devices (external catheter or urinal), as appropriate.
[00238] Impaired standing	[0202] Balance [0212] Coordinated Movement [0211] Skeletal function [2102] Pain Level	[5612] Teaching: prescribed exercise [140] Encouraging Body Mechanics [226] Exercise Therapy: Muscle Control [222] Exercise Therapy: Balance [224] Exercise Therapy: Joint Mobility [1806] Help with self-care: transfer	-Assist the patient with the initial ambulation, if necessary. -Instruct the patient/caregiver about safe transfer and ambulation techniques. -Control gait, balance and fatigue when walking. -Determine the limitations of joint movement and its effect on function. -Protect the patient from trauma during exercise.
[00303] Risk of adult falls	[1902] Risk Control [1912] Falls [1910] Safe Home Environment	[6490] Fall Prevention	-Identify cognitive or physical deficits of the patient that may increase the possibility of falls in a given environment. -Control gait, balance and fatigue when walking.
[00306] Risk of child falls	[1902] Risk Control [1912] Falls [1910] Safe Home Environment	[6490] Fall Prevention	-Identify cognitive or physical deficits of the patient that may increase the possibility of falls in a given environment. -Control gait, balance and fatigue when walking.
**Pattern 5: Sleep—Rest**			
**NANDA**	**NOC**	**NIC**	**ACTIVITIES**
[00095] Insomnia	[2002] Personal Wellness [2000] Quality of life	[5330] Mood Control [1850] Improve sleep [2300] Medication Administration	-Assess mood (signs, symptoms, personal history) initially and regularly as treatment progresses. -Determine the patient’s sleep/wake pattern. -Include the patient’s regular sleep/wake cycle in care planning. -Explain the importance of adequate sleep during pregnancy, illness, situations of psychosocial stress, etc. -Follow the five rules of proper medication administration.
[00198] Sleep pattern disorder	[0004] Dream	[1850] Improve sleep	-Determine the patient’s sleep/wake pattern. -Include the patient’s regular sleep/wake cycle in care planning.
**Pattern 6: Cognitive—Perceptual**			
**NANDA**	**NOC**	**NIC**	**ACTIVITIES**
[00126] Poor knowledge	[0907] Preparation of information	[5510] Health education	-Determine the personal context and sociocultural history of personal, family or community health behavior. -Determine the current health knowledge and lifestyle behaviors of the individuals, family or target group. -Help individuals, families and communities to clarify health beliefs and values.
[00132] Acute pain	[1605] Pain control [2102] Pain Level	[2210] Administration of analgesics [5820] Decreased anxiety [840] Position Change	-Check the medical orders regarding the medication, dose and frequency of the prescribed analgesic. -Check the patient’s previous response to analgesics (e.g., whether the non-opioid medication is as effective as the opiate). -Check previous doses and routes of administration of analgesics to avoid undertreatment or overtreatment. -Listen carefully. -Reinforce the behavior, as appropriate. -Create an environment that facilitates trust. -Place in the specified therapeutic position.
[00133] Chronic pain	[1605] Pain control [2102] Pain Level	[2210] Administration of analgesics [5820] Decreased anxiety [840] Position Change	-Check the medical orders regarding the medication, dose and frequency of the prescribed analgesic. -Check the patient’s previous response to analgesics (e.g., whether the non-opioid medication is as effective as the opiate). -Check previous doses and routes of administration of analgesics to avoid undertreatment or overtreatment. -Listen carefully. -Reinforce the behavior, as appropriate. -Create an environment that facilitates trust. -Place in the specified therapeutic position
[00214] Discomfort	[2008] State of Comfort	[6482] Environment Management: Comfort [5880] Relaxation Technique	-Determine patient and family goals for environmental manipulation and optimal comfort. -Prepare the transition of the patient and family by giving them a warm welcome to the new environment. -Create a quiet environment, without interruptions, with soft lights and a comfortable temperature, when possible.
**Pattern 7: Self-perception—Self -concept**			
**NANDA**	**NOC**	**NIC**	**ACTIVITIES**
[00124] Hopelessness	[1300] Acceptance: Health Status [1206] Desire to live [1204] Emotional Balance [1209] Motivation	[5330] Mood Control [5270] Emotional support [5230] Improve coping	-Assess mood (signs, symptoms, personal history) initially and regularly as treatment progresses. -Comment the emotional experience with the patient. -Explore with the patient what has triggered the emotions. -Make empathic or supportive affirmations. -Help the patient to solve problems constructively. -Assess the patient’s understanding of the disease process.
[00125] Impotence	[1702] Health beliefs: perception of control [1308] Adaptation to physical disability [1614] Personal autonomy	[5395] Improved self-confidence [5270] Emotional support	-Comment the emotional experience with the patient. -Explore with the patient what has triggered the emotions. -Make empathic or supportive affirmations. -Provide information about the desired behavior. -Help the individual commit to a plan of action to change behavior.
[00146] Anxiety	[1211] Anxiety Level [1402] Self-control of anxiety [0905] Concentration	[5820] Decreased anxiety [5230] Improve coping [6040] Relaxation therapy	-Listen carefully. -Reinforce the behavior, as appropriate. -Create an environment that facilitates trust. -Encourage the manifestation of feelings, perceptions and fears. -Identify changes in the level of anxiety. -Establish recreational activities aimed at reducing tensions. -Help the patient to identify situations that precipitate anxiety.
[00148] Fear	[1404] Fear Self Control [1210] Fear Level	[5820] Decreased anxiety [5230] Improve coping [5270] Emotional support	-Listen carefully. -Reinforce the behavior, as appropriate. -Create an environment that facilitates trust. -Encourage the manifestation of feelings, perceptions and fears.
[00153] Risk of situational low self-esteem	[1205] Self-esteem [1215] Self-awareness [1300] Acceptance: Health Status [1308] Adaptation to physical disability [1302] Coping with problems [1614] Personal autonomy	[5400] Enhancement of self-esteem [5270] Emotional support [6400] Support in protection against abuse [5240] Advice	-Determine the patient’s confidence in their own criteria. -Encourage the patient to identify their strengths. Help the patient find self- acceptance. -Determine if the child/dependent adult is viewed differently by an adult based on sex, appearance, or behavior. -Identify crisis situations that may trigger abuse, such as poverty, unemployment, divorce or death of a loved one.
**Pattern 8: Role—Relationships**			
**NANDA**	**NOC**	**NIC**	**ACTIVITIES**
[00051] Impaired verbal communication	[0902] Communication [0907] Preparation of information	[4920] Listen Active [4974] Improve communication: hearing impairment [4976] Improve communication: speech deficit	-Show interest in the patient. -Ask questions or statements that encourage expressing thoughts, feelings and concerns. -Carry out or organize routine hearing evaluations and screenings. -Monitor the speed, pressure, rhythm, amount, volume and diction of speech. -Monitor the cognitive, anatomical, and physiological processes associated with speech capabilities (e.g., memory, hearing, and language). -Instruct the patient or family about the cognitive, anatomical and physiological processes involved in speech abilities.
[00062] Risk of caregiver role fatigue	[2205] Primary Caregiver Performance: Direct Care [2206] Primary caregiver performance: indirect care	[7040] Primary Caregiver Support	-Determine the level of knowledge of the caregiver. -Determine the caregiver’s acceptance of their role. -Encourage the caregiver to participate in support groups. -Teach the caregiver health care maintenance strategies to promote their own physical and mental health.
**Pattern 9: Sexuality and Reproduction**			
**NANDA**	**NOC**	**NIC**	**ACTIVITIES**
[00227] Risk of ineffective maternity process	[1908] Risk Detection [2013] Balance in lifestyle	[5440] Increase Support Systems	
**Pattern 10: Adaptation—Stress Tolerance**			
**NANDA**	**NOC**	**NIC**	**ACTIVITIES**
[00177] Overload stress	[1212] Stress Level [1308] Adaptation to physical disability	[8340] Foster resilience [5230] Improve coping [5270] Emotional support	-Promote family support. -Facilitate family communication. -Help the patient develop an objective assessment of the event. -Make empathic or supportive affirmations. -Hug or touch the patient to provide support.
**Pattern 11: Values—Beliefs**			
**NANDA**	**NOC**	**NIC**	**ACTIVITIES**
[00066] Spiritual suffering	[1300] Acceptance: Health Status [1302] Coping with problems [1215] Self-awareness	[5426] Facilitate spiritual growth [5270] Emotional support [5250] Support in decision making [5240] Advice	-Show assistance and comfort by spending time with the patient, with the patient’s family and with those close to them. -Encourage conversation that helps the patient organize spiritual interests. -Model healthy relationship and reasoning skills. -Make empathic or supportive affirmations. -Hug or touch the patient to provide support.

## Appendix B. Autonomy Problems

**Problems of Autonomy**		
**NEED**	**NIC**	**ACTIVITIES**
Feeding	[1803] Help with self-care: feeding	-Control the patient’s ability to swallow. -Create a pleasant environment during mealtime (place bedpans, urinals and vacuum equipment out of sight). -Ensure the proper position of the patient to facilitate chewing and swallowing. Provide physical help, if needed. -Provide devices adapted to facilitate self-feeding (long handles, handles with a large circumference, or small straps on utensils), if necessary.
Elimination	[1804] Help with self-care: urination/defecation	-Assist the patient on the toilet/portable toilet/fracture wedge/urinal at specified intervals. -Provide privacy during elimination. -Provide assistive devices (external catheter or urinal), as appropriate.
Mobilization	[1806] Help with self-care: transfer	-Determine the patient’s current ability to transfer independently (e.g., level of mobility, movement limitations, endurance, ability to stand and bear weight, medical or orthopedic instability, level of consciousness, ability to cooperate, ability to understand instructions). -Teach the patient all the appropriate techniques in order to achieve the maximum level of independence. -Teach the individual the techniques of transferring from one area to another (e.g., from bed to chair, from wheelchair to vehicle). -Teach individual the use of ambulatory aids (e.g., crutches, wheelchair, walkers, trapeze bar, cane).
Dress and Personal Grooming	[1802] Help with self-care: dressing/grooming	-Maintain privacy when the patient is dressed. -Be available to help with dressing, if needed. -Reinforce efforts to dress alone.
Maintenance of Body Temperature	[3900] Temperature regulation.	-Observe the color and temperature of the skin. -Observe and record if there are signs and symptoms of hypothermia and hyperthermia. -Adjust the room temperature to the needs of the patient. -Teach the patient to avoid heat exhaustion and heat stroke.
Hygiene and care of the skin, mucous and fanera.	[1801] Help with self-care: bathing/hygiene	-Control the skin integrity of the patient. -Maintain hygienic rituals. -Facilitate the maintenance of the patient’s routines at bedtime, signs of sleep onset and familiar objects (for children their favorite blanket or toy, rocking, pacifier or story; for adults read a book or have a pillow from home), as appropriate. -Encourage the participation of parents/family in the usual rituals at bedtime, if applicable. -Provide help until the patient is fully capable of self-care.
Safety Maintenance of the Environment	[1805] Help with self-care: IADL	-Determine the individual’s need for help with instrumental activities of daily living (e.g., shopping, cooking, housework, laundry, using public transportation, managing money, managing medications, communicating, and time management)). -Determine needs for changes related to safety in the home (e.g., widen door frames to allow wheelchair access to bathroom, remove rugs). -Determine home improvement needs to counteract disabilities (e.g., put large numbers on phone, turn up phone ring volume, move washer and other appliances to main floor, put side rails on hallway, grab bars in bathrooms).

## Appendix C. Collaboration Problems

**Collaboration Problems**			
**PROFESSION**	**OBSERVATIONS**	**NIC**	**ACTIVITIES**
Medicine	The medical implication is indisputable for the approach of this syndrome. The medical vision provides the necessary perspective for multidisciplinary treatment. The phenotype and the variable symptoms of the pathology require a medical study by different specialties and subsequently a pooling to achieve a complete medical assessment. The prescription and analysis of diagnostic tests, treatment and possible surgical interventions derived from the disease process are the main collaborative links with this healthcare group.	[2300] Medication administration [2395] Medication control. [2900] Surgical assistance. [7320] Case management. [7610] Diagnostic tests at the point of care. [7680] Help in exploration. [7690] Interpretation of laboratory data. [8020] Multidisciplinary care meeting	-Follow the five rules of proper medication administration. -Predict and provide the necessary supplies and instruments during the procedure. -Ensure that appropriate instruments, supplies, and equipment are sterile and in good working order. -Develop relationships with the patient, family, and other healthcare providers, as needed. -Use effective communication skills with the patient, family and other health care providers. -Record the results of the tests, in accordance with the institutional procedure. -Verify the results of the analytics performed at the point of care with a central laboratory when a critical clinical decision is to be made. -Inform the doctor about abnormal or critical results, as appropriate. -Explain to the patient each step of the procedure. Monitor the patient’s condition during the procedure. Provide emotional support to the patient, if indicated. -Facilitate communication and collaboration between members of the multidisciplinary team to ensure effective and focused discussions that allow team members to solve problems and efficiently provide patient needs.
Psychology	Patients diagnosed with this syndrome are associated with a great psychological burden due to the setting in which the pathology develops. The uncertainty about the future, the lack of knowledge of their disease, the delay in diagnosis, the lack of effective treatment and the high invalidating, degenerative and dependent potential generate mental disorders that require psychological care. Anxiety, stress and depression are the most frequent mental disorders derived from this disease.	[5395] Improved self-confidence [5400] Enhancement of self-esteem. [5820] Decreased anxiety. [5270] Emotional support. [5450] Group therapy	-Encourage the patient to identify their strengths. Help the patient find self- acceptance. -Determine if the child/dependent adult is viewed differently by an adult based on sex, appearance, or behavior. -Identify crisis situations that may trigger abuse, such as poverty, unemployment, divorce or death of a loved one. -Listen carefully. -Reinforce the behavior, as appropriate. -Create an environment that facilitates trust. -Encourage the manifestation of feelings, perceptions and fears. -Identify changes in the level of anxiety. -Establish recreational activities aimed at reducing tensions. -Help the patient to identify situations that precipitate anxiety. -Choose group members who are willing to actively participate and take responsibility for their own problems. -Determine if the level of motivation is high enough to benefit from group therapy.
Physiotherapy	musculoskeletal involvement and its impact on the mobility of these patients requires rehabilitation care. Maintaining muscle tone, caring for joints, pain, and respiratory physiotherapy are key in caring for this syndrome.	[0200] Promotion of exercise [0201] Exercise promotion: strength training [0202] Promotion of exercise: stretching [0221] Exercise therapy: ambulation [0222] Exercise therapy: balance [0224] Exercise therapy: joint mobility [0226] Exercise therapy: muscle control	-Assist the patient with the initial ambulation, if necessary. -Instruct the patient/caregiver about safe transfer and ambulation techniques. -Control gait, balance and fatigue when walking. -Determine the limitations of joint movement and its effect on function. -Protect the patient from trauma during exercise. -Consult with the physical therapist about the ambulation plan, if necessary.
Chiropody	Acroosteolysis of the distal phalanges of the feet and hands with their corresponding deformity. Shortened and thick fingers and watch glass nails require specific care for this health group.	[1660] Foot care [1680] Nail care. [0221] Exercise therapy: ambulation	-Inspect if there is irritation, cracks, lesions, calluses, deformities or edema in the feet. -Inspect the patient’s shoes to see if they fit correctly. -Soak your feet, if necessary. -Carefully dry the interdigital spaces. -Apply lotion. -Clean nails. -Apply/provide an assistive device (cane, crutches, or wheelchair, etc.) for ambulation if the patient is unstable. -Assist the patient with the initial ambulation, if necessary. -Instruct the patient/caregiver about safe transfer and ambulation techniques.
